# A prospective randomised controlled trial of operative *versus* non-operative management of fractures of the humeral diaphysis: the HUmeral Shaft Fracture FIXation (HU-FIX) Study protocol

**DOI:** 10.1186/s13063-019-3576-0

**Published:** 2019-08-05

**Authors:** William M. Oliver, Thomas H. Carter, Catriona Graham, Timothy O. White, Nicholas D. Clement, Andrew D. Duckworth, Samuel G. Molyneux

**Affiliations:** Edinburgh Orthopaedic Trauma, Royal Infirmary of Edinburgh, 51 Little France Crescent, Edinburgh, Midlothian, EH16 4SA UK

**Keywords:** Humerus, Humeral, Shaft, Diaphysis, Fracture, Randomised controlled trial, Operative, Fixation, Non-operative, Bracing, Trauma, Patient outcome

## Abstract

**Background:**

Humeral shaft fractures constitute around 1% of adult fractures in the UK, with an annual incidence of approximately 13 per 100,000 population. Historically, these injuries have been primarily managed non-operatively, with operative fixation reserved for specific indications. Although some recent retrospective studies have suggested there are potential benefits of operative fixation over humeral bracing, there is a deficiency in level 1 evidence to support operative management as the primary treatment for humeral shaft fractures.

**Methods/design:**

This single-centre prospective randomised controlled trial aims to recruit 70 adult patients with an isolated closed fracture of the humeral diaphysis into one of two treatment arms: operative (*n* = 35) or non-operative (*n* = 35). The operative arm will undergo open reduction and internal fixation (ORIF) of the fracture using a standard fixation technique (plate and screws). The non-operative arm will be fitted with a prefabricated humeral brace until fracture union. All patients will be followed up for 1 year post-intervention. The primary outcome measure will be the Disabilities of the Arm, Shoulder and Hand (DASH) score at 3 months post-intervention. Secondary outcome measures will include pain, treatment complications, return to work or sporting activities, shoulder and elbow range of motion, radiographic assessment, EuroQol (EQ-5D) Health Outcome score and 12-item Short Form (SF-12) Health Survey score. A health economic analysis will be performed to compare the cost implications of each treatment strategy.

**Discussion:**

This randomised controlled trial will provide level 1 evidence comparing a standard ORIF technique against functional bracing for isolated closed humeral shaft fractures. The investigators hope that the study results will assist surgeons in their decision-making when managing patients with these injuries.

**Trial registration:**

ClinicalTrials.gov, NCT03689335. Registered on 28 September 2018 (retrospectively).

**Electronic supplementary material:**

The online version of this article (10.1186/s13063-019-3576-0) contains supplementary material, which is available to authorized users.

## Background

Fractures of the humeral diaphysis (humeral shaft fractures) constitute 1.2% of adult fractures in the UK, with an overall incidence of 12.9 per 100,000 per year [1]. These injuries are more common in women and have a bimodal age distribution, with peaks of prevalence occurring in younger adults following high-energy trauma and older adults following low-energy trauma [1–3].

Humeral shaft fractures are routinely managed non-operatively, with the widespread use of a prefabricated humeral brace, as first described by Sarmiento et al*.* in 1977 [4]. The purported benefits of this technique (over conventional plaster immobilisation) include rapid and uninterrupted osteogenesis and early mobilisation of the shoulder and elbow, thus enhancing functional outcomes [4]. Subsequent studies comparing bracing with plaster immobilisation have demonstrated faster fracture union, reduced rates of varus mal-alignment and confirmed functional advantages in terms of shoulder and elbow mobility [5, 6].

Although functional bracing has resulted in low rates of humeral shaft fracture nonunion in some series [7–9], others have reported nonunion rates of 10% or more [10–13], with the highest rate reported as 33% [14]. When fracture union does occur, the reported time to union ranges from 7.5 to 11.5 weeks [7, 9], during which time almost all activities of daily living are restricted, and there is a risk of skin breakdown and secondary cellulitis that can necessitate brace removal [13]. Moreover, while malunion is considered to be tolerated well by patients, the acceptable thresholds are based upon an historic series of just 32 patients, who were examined by a single author [15]. A more recent series has suggested that malunion and consequent loss of shoulder range of motion can occur in up to 38% of patients treated with a brace [16].

The perceived limitations of humeral bracing have brought about a gradually increasing role for operative fixation [17]. Although intramedullary nailing is considered feasible, published case series [18], reviews [19, 20] and randomised controlled trials [21, 22] have consistently recommended open reduction and internal fixation (ORIF) as the strategy of choice when surgery is indicated.

The primary aim of this trial is to determine whether any functional difference exists, according to the Disabilities of the Arm, Shoulder and Hand (DASH) score at 3 months post-intervention, between operative (ORIF) and non-operative management (functional bracing) of humeral shaft fractures. The secondary aim of this trial is to determine whether any difference exists in other clinically important outcomes (including pain, treatment complications, return to work or sporting activities, shoulder and elbow range of motion, fracture union, patient-reported outcome measures and economic costs) between ORIF and functional bracing of humeral shaft fractures in the year following the intervention.

## Methods/design

The Humeral Shaft Fracture Fixation (HU-FIX) Study is a single-centre prospective randomised controlled trial that aims to assess whether there is any difference in outcome between patients with a humeral shaft fracture who are managed operatively (i.e. with surgical fixation) and those managed non-operatively. The study will adhere to the principles in the latest Consolidated Standards of Reporting Trials (CONSORT) Statement [23]. The study will be performed at a large tertiary referral centre. The study received a favourable opinion from the relevant research ethics committee on 19 July 2018 (South-East Scotland Research Ethics Committee 01 reference 18/SS/0073). The study is sponsored by National Health Service (NHS) Lothian Research and Development and was approved on 10 September 2018 (project number 2018/0223). The study was registered with the ClinicalTrials.gov database, which is operated by the US National Library of Medicine, on 28 September 2018 (ID NCT03689335). The study is funded and co-sponsored by the Scottish Orthopaedic Research Trust into Trauma (SORT-iT).

**Fig. 1:**
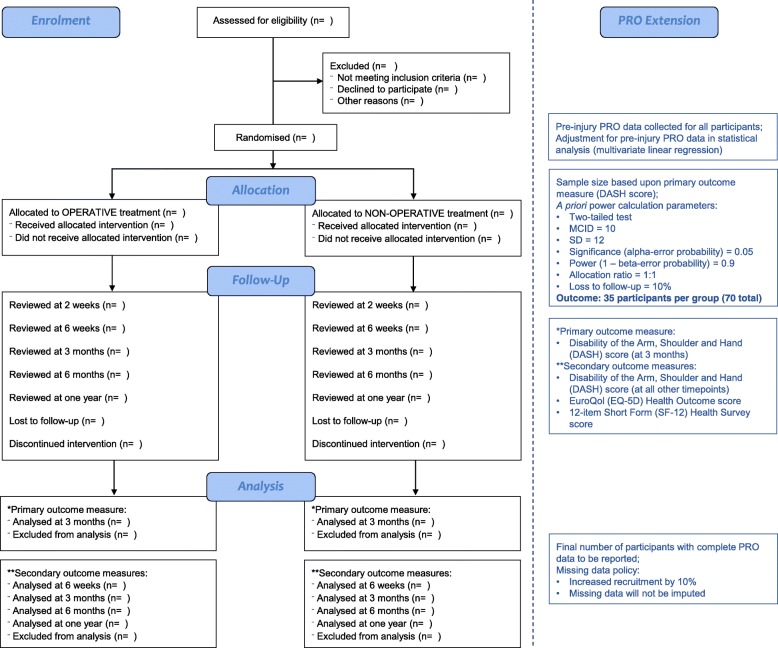
CONSORT flow diagram (with PRO extension) for the HU-FIX study. HU-FIX Humeral Shaft Fracture Fixation

The trial will include all eligible patients presenting to the study centre with an isolated closed fracture of the humeral shaft. The planned flow of patients through the study is as shown in Fig. 1.

**Fig. 2:**
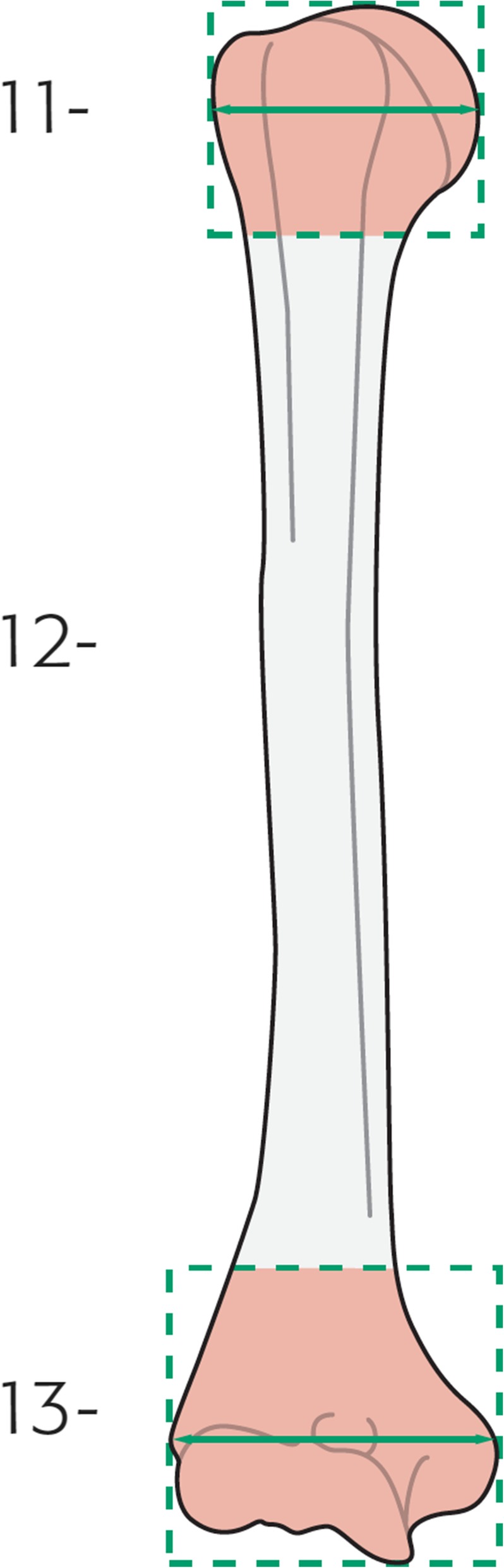
*Arbeitsgemeinschaft für Osteosynthesefragen* definition of humeral shaft (diaphyseal segment, 12-), with Müller boxes delineating the proximal (11-) and distal (13-) segments

A fracture of the humeral shaft (diaphyseal segment) is defined as any humeral fracture in which the major fracture line does not extend to within one metaphyseal width (Müller box) of either the shoulder or elbow joint; this is consistent with the *Arbeitsgemeinschaft für Osteosynthesefragen* definition [24], and is illustrated in Fig. 2.

### Inclusion criteria

1. Fracture of the humeral shaft
2. Closed injury
3. Age ≥ 16 years
4. Able to provide informed consent in English
5. Surgery performed within 3 weeks of date of injury

### Exclusion criteria

1. Completely undisplaced fractures
2. Injuries considered to be an absolute indication for surgery (including severe associated neurovascular injury, open fractures and bilateral injuries)
3. Patients with a periprosthetic or pathological fracture
4. Patients with an additional spine or limb injury (including those with polytrauma), which may impact upon functional rehabilitation
5. Patients medically unfit for surgery
6. Very frail patients, defined as those with a Clinical Frailty Score of ≥6/9 as described in the Canadian Study on Health and Aging [25]
7. Pregnant women with predetermined treatment
8. Patients declining operative management
9. Patients unable to comply with post-operative data gathering, including completing questionnaires in English
10. Non-residents or those unable to return to the Unit for the 1-year follow-up period
11. Patients for whom the treating surgeon does not feel that inclusion in the trial is in their best interests, either due to fracture pattern or patient factors

### Enrolment and allocation

Potential HU-FIX study participants will be identified at the point of referral to the Orthopaedic Service, either directly (via the on-call team) or via the Trauma Triage Clinic system [26]. Patients satisfying the inclusion and exclusion criteria will be introduced to the study by the treating clinical team and provided with a patient information sheet to read before being asked to participate. Patients will be predominantly enrolled into the study either on the Orthopaedic Ward (if admitted) or at their first outpatient clinic appointment. A study investigator will review the study protocol in detail with the patient and address any of their questions. If the patient is willing to participate, a study investigator will complete the informed consent process.

Once informed consent has been obtained, patients will be enrolled into the study by an investigator and randomly allocated to either operative or non-operative management of their humeral shaft fracture.

### Randomisation

The study will use a parallel assignment model, with 1:1 allocation of patients into each treatment arm (operative or non-operative). Randomisation will be stratified by patient age (< 65 years old or ≥ 65 years old), to ensure there are approximately equal numbers of younger and older patients in each treatment arm. Using block randomisation with a mixed block size and a 1:1 ratio of operative to non-operative, a computer-generated randomisation schedule was produced by a senior statistician from the local clinical research facility using nQuery Advisor v7.0 software (Statsols, Cork, Ireland). A member of staff independent of the study used this schedule to produce a series of opaque sealed envelopes, each containing a sticker bearing the words ‘operative’ or ‘non-operative’. Once the randomisation envelope is opened, the sticker is placed onto the already-signed consent form to indicate the treatment group allocation.

### Interventions

All patients will be initially treated in the Emergency Department with closed reduction of their humeral shaft fracture, and application of a either a U-slab or above-elbow hanging plaster cast.

Patients allocated to *operative* management of their humeral shaft fracture will undergo surgical fixation, using a standard technique of plating and screw fixation. The exact surgical approach and fixation technique utilised will be at the discretion of the treating surgeon. Similarly, post-operative immobilisation and range-of-motion restrictions will be at the discretion of the treating surgeon. This is determined by a number of factors including the injury and fracture pattern, bone quality, co-morbidities and patient compliance. The approach, fixation technique and range-of-motion restrictions will be recorded for all patients and presented as a sub-group analysis. Although the study sample will not be specifically powered on this basis (see Power calculation and statistical analysis, below), the investigators hope that a sub-group analysis will nonetheless allow any differences between the groups to be detected.

Patients allocated to *non-operative* management will be managed as per current standard practice for the study centre. This involves a period of immobilisation in a U-slab or hanging cast for up to 2 weeks, before fitting with a lightweight prefabricated humeral brace in the outpatient clinic. Two specific models of humeral brace are in routine use in this centre: the Clasby humeral brace (Beagle Orthopaedic, Blackburn, UK) and the ProCare over-the-shoulder humeral fracture brace (DJO Global, Vista, CA). The current standard of care in the study centre is to use the Clasby brace for proximal-third or mid-shaft fractures and the ProCare brace for distal-third or multifragmentary fractures. Brace selection for study participants will reflect these indications but will remain at the treating surgeon’s discretion. Patients will be permitted to start passive pendular shoulder exercises with full elbow, wrist and hand mobilisation as soon as the humeral brace has been applied. Post-injury physiotherapy will be arranged at the discretion of the treating surgeon, as occurs in everyday clinical practice.

### Outcome assessment

All follow-up assessments will take place during outpatient clinic visits at 2 weeks, 6 weeks, 3 months, 6 months and 1 year. At each clinic visit, a physical examination will be performed, complications noted and the need for any further surgery recorded. Patients will be given time to complete a self-reported questionnaire, which will generate clinical outcome scores. The 1-year follow-up will enable study investigators to assess whether any differences between the operative and non-operative groups (if any) are sustained for 1 year post-intervention and to detect any important complications that may occur within the first year (e.g. nonunion).

**Fig. 3:**
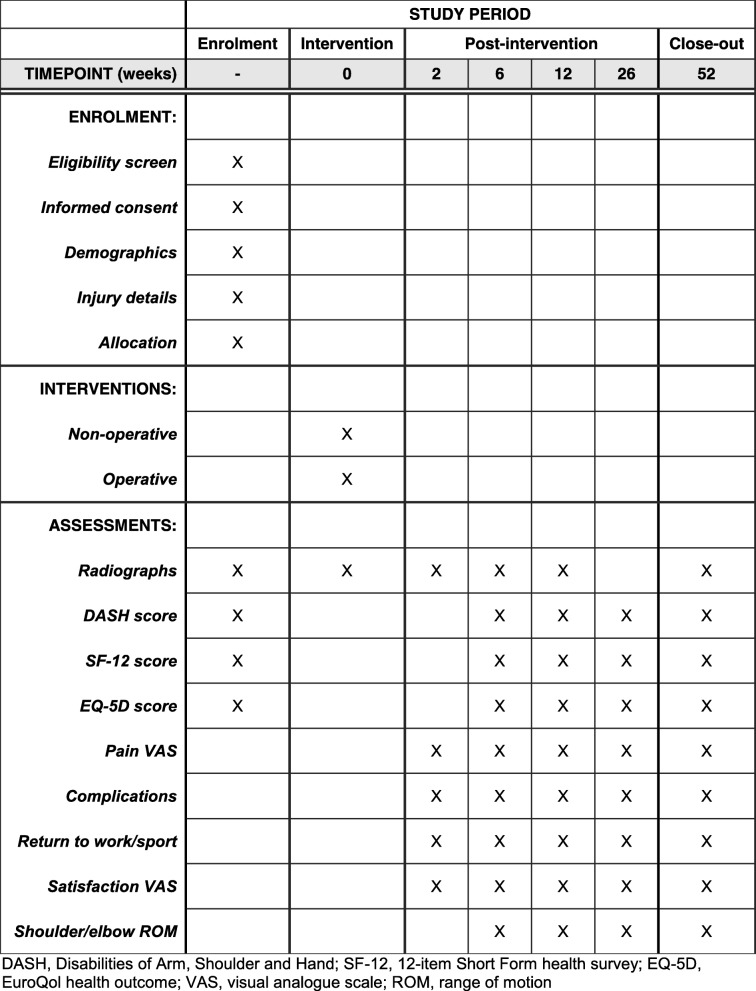
SPIRIT figure for the HU-FIX study. DASH Disabilities of the Arm, Shoulder and Hand, EQ-5D EuroQol Health Outcome, ROM range of movement, SF-12 12-item Short Form Health Survey, VAS visual analogue scale

The Standard Protocol Items: Recommendations for Interventional Trials (SPIRIT) [27] checklist is given in Additional file 1. The SPIRIT schedule of enrolment, interventions and assessments is shown in Fig. 3.

### Primary outcome measure

The primary outcome measure is the DASH score at 3 months post-intervention. The DASH score is a functional rating scale that was developed in 1996. It has been used extensively as a research tool in upper limb surgery [28]. It consists of a 30-item self-reported questionnaire, and two optional modules, one assessing work and the other sports and performing arts. Patient symptoms (including pain, weakness, stiffness and tingling or numbness) and functional status (including physical, social and psychological aspects) during the week prior to survey completion are assessed. The final score is from 0 to 100, with 0 representing no disability and 100 the worst possible disability. A recent study demonstrated the DASH score to be both valid and reliable in evaluating outcomes in patients following a humeral shaft fracture [29].

The null hypothesis is that there is *no* *difference* in early functional outcomes (DASH score at 3 months post-intervention) between the operative and non-operative management of patients following a humeral shaft fracture.

### Secondary outcome measures

Secondary outcome measures will include:

1. Change in the DASH score over the 1-year follow-up period
2. Pain assessment, using a visual analogue scale (0 to 10)
3. Complications of treatment, including neurovascular injury, superficial or deep infection, failure of fixation, revision surgery, skin complications (including eczema and cellulitis) and complex regional pain syndrome
4. Time to return to work and sports (if relevant)
5. Satisfaction with the service, using a visual analogue scale (0 to 100), and with the appearance of the affected limb, also using a visual analogue scale (0 to 10)
6. Range of motion at the shoulder and elbow
7. Radiographic assessment, using standard anteroposterior and lateral radiographs of the humerus to assess the progression of fracture healing (union or nonunion) and final humeral deformity (malunion)
8. EuroQol (EQ-5D) health outcome score [30]
9. 12-item Short Form (SF-12) health survey score [31]
10. Health economic analysis

Outcome measures 1 to 9 have been collated into a single data collection form (one form per study participant).

### Power calculation and statistical analysis

Prior to study commencement, a prospective power calculation was used to determine the number of patients required in each treatment group. The minimal clinically important difference in the DASH score is reported to be 10 points [32], and the assumed standard deviation is 12 points for both the operative and non-operative groups [33]. Thus, using a two-sided *t*-test with 5% significance, a total of 70 patients (35 in each group) are required to produce a 90% power to detect a meaningful difference (in terms of the DASH score at 3 months post-intervention) between the two groups, assuming a 10% loss to follow-up.

Data will be analysed on an intention-to-treat basis. Data analysis will be performed by a senior statistician, employed by the local clinical research facility and independent of the study team. The primary outcome measure (DASH score at 3 months post-intervention) will be compared between the two treatment arms, using a two-sided independent samples *t*-test or non-parametric equivalent (as indicated by the normality of the data). This method will also be used to compare the other continuous outcome measures between the two treatment arms (e.g. pain score, satisfaction scores, shoulder and elbow range of motion, EQ-5D score and SF-12 score). The pattern of change in continuous outcome measures over the study period will be presented graphically, broken down by treatment allocation. A linear regression model will be generated for each participant by plotting the DASH score from 6 weeks to 1 year post-intervention and the gradient of the regression line (i.e. the rate of change in the score) will be compared between treatment groups. Multivariate linear regression, including analysis of covariance (ANCOVA), will also be used to adjust for the potential effect of pre-injury DASH score, age and other prognostic factors affecting the DASH score. Binary outcomes (e.g. treatment complications, re-operation and nonunion or malunion) will be compared between the two treatment arms using a binomial test for the comparison of proportions. Two-tailed *p* values will be reported where possible, and statistical significance will be set at *p* = 0.05.

### Missing data

The study sample size has been increased to provide for up to 10% loss to follow-up at the primary outcome timepoint (3 months post-intervention). All clinical and outcome data will be collected in the outpatient clinic, where clinical reviews and radiographs (where required) are also performed. These appointments reflect routine follow-ups of a humeral shaft fracture in the study centre, and the investigators anticipate this arrangement will limit missing data considerably. The participant dropout rate for each treatment arm will be reported and compared. If the participant dropout rate at the primary outcome timepoint is substantial, a sensitivity analysis may be performed. Missing data at other timepoints will be accounted for by plotting a line of best fit for the data points for each participant and comparing the gradient of the regression line (i.e. the rate of change in the variable of interest) between treatment groups.

### Health economic analysis

Direct costs of the injury and its treatment will be assessed, including the costs for any of the following:

- plaster or brace immobilisation
- any surgical procedure (including implant costs, theatre time, hospital admission and treatment of early complications)
- analgesia or other treatments for ongoing pain or other complications (pharmaceutical or otherwise)
- any additional clinical input (including primary and secondary care encounters and re-admissions to hospital)
- any allied health professional input (including physiotherapy and occupational therapy)
- any occupational or statutory sick pay received during employment absence

Secondary costs will also be assessed, including:

- any loss of earnings
- any additional support required for patients to complete their activities of daily living or to fulfil their own caring responsibilities to others

These data will allow an analysis of the overall economic impact of humeral shaft fractures and a comparison of the cost implications of each treatment strategy. A cost per quality-adjusted life year (QALY) gained will be calculated (total cost difference between treatment groups / difference in QALYs between treatment groups). QALYs will be calculated at 1 year, using the difference between the EQ-5D scores at the follow-up intervals.

### Patient involvement, safety and confidentiality

Patient and public involvement was not sought for this small, single-centre trial.

The maximum possible radiation dose for study participants is equivalent to 2 weeks of the dose to the UK population from natural sources of background radiation. The risk of fatal cancer arising from this level of exposure is negligible, at 1 in 230,000 [34]. This proposed radiation exposure has been approved by a medical physics expert and a consultant radiologist.

Complication rates will be monitored by study investigators throughout the trial, but a formal data monitoring committee will not be convened. Both treatment options (operative and non-operative) are regularly employed in the study centre, and any additional risk to patient safety is low. The study sponsor will be regularly updated with study progress and any patient safety issues that may arise, including adverse or serious adverse events. The trial steering committee will be consulted if required. The procedure for identifying, recording and reporting adverse events and urgent safety measures will adhere to relevant guidelines from the study sponsor, which will also advise upon any action required to protect patient safety.

Data collection forms will be kept in a secure filing cabinet in the research office on the study site, both of which are locked when not in use by the study investigators. These personal data will then be entered and stored electronically, under a password-protected file on a password-protected NHS computer system and will be accessible only by the study investigators. Once data collection is complete and analysis begins, non-personal data (stored using unique participant numbers rather than names or other identifiable information) will be made available to other authorised members of the research team. All electronic data will be handled according to study sponsor guidelines on data protection and confidentiality.

When finalised, study results will be disseminated via an internal report, conference presentations and peer-reviewed scientific journals. A summary of the results will be made available to study participants upon request.

## Discussion

A review of the existing literature indicates that there is a paucity of level 1 evidence to support operative management as the primary treatment for humeral shaft fractures. Several studies have retrospectively compared humeral shaft fracture outcomes following non-operative and operative management. Jawa et al*.* compared complication rates for distal-third humeral shaft fractures treated with bracing versus those treated with ORIF, concluding that both strategies had potential risks and that the optimal treatment was based on patient preference [35]. More recently, Denard et al*.* demonstrated that there was an increased likelihood of both nonunion and malunion with bracing, and in the context of comparable (or lower) complication rates with ORIF, concluded that ORIF should be performed more frequently [36]. Mahabier et al*.* also suggested there was little difference between non-operative and operative treatment in terms of fracture union or nerve injury [37].

In the only published randomised controlled trial of which the authors are aware, Matsunaga et al*.* compared bracing with a less widely employed minimally invasive bridge plating technique, demonstrating a statistically (but not clinically) significant difference in the DASH score in favour of the fixation group, along with significantly lower rates of nonunion, contact dermatitis and coronal plane malalignment at 6 months in the fixation group [38]. There was no significant difference in the DASH score at any other timepoint, and no difference in any other functional outcomes between the groups. However, this study did not assess the current gold standard surgical technique of compression plating and included a population substantially younger than the average patient sustaining a humeral shaft fracture in the UK [1].

Two ongoing randomised controlled trials comparing surgical fixation against functional bracing are currently recruiting patients, one in Canada (ClinicalTrials.gov ID NCT00878319) and one in Finland (ClinicalTrials.gov ID NCT01719887) [39]. A further prospective multi-centre observational study comparing functional recovery after operative versus non-operative treatment is also underway in the Netherlands (Netherlands Trial Register ID NTR3617) [40]. The HU-FIX study aims to contribute to this growing body of literature and provide greater clarity to surgeons managing patients with humeral shaft fractures.

The study is pragmatic and allows for normal variations in clinical practice, including in the surgical approach and fixation method used during ORIF, as well as the involvement of physiotherapy. The investigators hope that this pragmatic design will reproduce day-to-day trauma care and improve the external validity of study results [41, 42]. Potential limitations include the single-centre study design and the lack of blinding. Edinburgh Orthopaedic Trauma provides trauma care to a catchment population of approximately 850,000 and consists of 12 consultant orthopaedic trauma surgeons who collectively treat approximately 70 humeral shaft fractures per year [1]. The investigators are, therefore, confident that the study results can be reliably extrapolated to the wider orthopaedic community. Patient blinding is not possible in this study given the nature of the interventions being compared, neither is assessor blinding, as the presence or absence of a surgical scar or metalwork on plain radiographs will be obvious during outcome assessment. Again, this is consistent with a pragmatic design that reflects everyday clinical practice. Although this study is not a pilot for a larger multi-centre randomised controlled trial, the investigators hope that the results of the HU-FIX Study will inform researchers planning or preparing a multi-centre trial in the future.

Use of patient-reported measures to assess outcomes after a traumatic injury is consistently expanding [43], with the main advantage being that determining injury outcome or treatment success is increasingly becoming patient-centred. The DASH score is a holistic measure of outcome, assessing impairment, activity limitation and participant restriction [44]. However, it does not distinguish between the injured and uninjured limbs, which may artificially inflate scores for patients who are more able to compensate for the loss of function in one limb (e.g. young active patients with injuries to their non-dominant arm). It is, therefore, important to adjust for these potential confounding factors during statistical analysis.

### Trial status

This manuscript is based on HU-FIX study protocol version 1.3 (29 August 2018). The first patient was enrolled on 19 September 2018. The investigators estimate enrolment will be complete by July 2020, with the study follow-up complete by July 2021.

## Additional file

Additional file 1: SPIRIT 2013 Checklist: Recommended items to address in a clinical trial protocol and related documents. (DOC 127 kb)

## Data Availability

Study data will be made available upon request.

## Abbreviations

CONSORT: Consolidated Standards of Reporting Trials
DASH: Disabilities of the Arm, Shoulder and Hand
EQ-5D: EuroQol Health Outcome
HU-FIX: Humeral Shaft Fracture Fixation
NHS: National Health Service
ORIF: Open reduction and internal fixation
QALY: Quality-adjusted life year
ROM: Range of movement
SF-12: 12-item Short Form Health Survey
SORT-iT: Scottish Orthopaedic Research Trust into Trauma
SPIRIT: Standard Protocol Items: Recommendations for Interventional Trials
VAS: Visual analogue scale
